# Beyond the ivory tower: Measuring and explaining academic engagement with journalists, politicians and industry representatives among Swiss professors

**DOI:** 10.1371/journal.pone.0251051

**Published:** 2021-05-21

**Authors:** Adrian Rauchfleisch, Mike S. Schäfer, Dario Siegen

**Affiliations:** 1 Graduate Institute of Journalism, National Taiwan University, Taipei, Taiwan, R.O.C; 2 Department of Communication and Media Research, University of Zurich, Zurich, Switzerland; Universidad de las Palmas de Gran Canaria, SPAIN

## Abstract

Scholars from different theoretical schools have posited that in recent decades, science and society have moved closer together, and the concept of academic engagement has been proposed to capture one part of this approximation empirically. This study analyzes the academic engagement of individual scholars towards politicians, industry representatives and journalists. It uses comprehensive survey data on Swiss professors from all disciplines, all the country’s universities and from associated research institutes. It assesses, firstly, the degree to which these professors have professional contacts to journalists, politicians and industry representatives. Secondly, it explains the extent of these contacts, using multi-level modelling that incorporates individual factors as well as organizational and institutional contexts. Our study shows that academic engagement is quite common with strong differences between disciplines. Furthermore, professors with higher academic productivity, positive personal attitude towards communication activities as well as a leadership position have more outside contacts. The gender and nationality of the professors, however, only play a role for some of the contacts with non-scientific actors.

## 1. Introduction

Scholars from different disciplines–including the social sciences and humanities–have gone beyond the proverbial academic "ivory tower" in recent years. They attend political hearings like US atmospheric scientist Michael E. Mann or Swiss climatologist Thomas Stocker, publish columns in news media like Nobel laureate and economist Paul Krugman, or cooperate with global corporations like Indian computer scientist Rajeev Motwani.

More examples for exchanges between scientists and politicians, industry representatives and journalists could be found–and this anecdotal evidence is backed up by social theorists observing a general approximation between science and other social realms, as well as between the respective organizations and individuals [1–4]. Economists [5–7], innovation researchers [2, 8], sociologists [9–11] and communication scholars [12, 13], among others, have argued that science and other realms of society have become tighter coupled in recent decades. Most of these conceptual diagnoses, from earlier "mode 1/mode 2" [14] discussions over "contextualization" [11] and "societalization" [9, 10] to descriptions of a "triple" [1] or "quadruple helix" [15], focus on science’s relation to politics and the economy [2, for overviews 3], and more recently, also to the news media [10, 15].

To capture these linkages analytically, Perkmann et al. [4] have proposed the concept of *academic engagement*, originally developed for the science-industry nexus but applicable beyond that. Using a broad and inclusive analytical vista [see also 2], the concept aims to capture all "knowledge-related collaboration[s] by academic researchers with non-academic organizations" including "collaborative research, contract research, and consulting, as well as informal activities like providing ad hoc advice and networking with practitioners" [4].

These diagnoses have inspired a considerable number of empirical studies, which have mostly focused on the organizational and individual linkages between science and the economy [2, for overviews 3], but also, albeit less often, between science and politics [for overviews 16, 17], and science and the news media [12, 18]. The respective research has described the extent of such linkages, as well as different antecedents and drivers of such contacts. But the field still has a number of rather understudied areas: Only few studies have compared engagement with different non-academic organizations or individuals and their explanatory models have not always used multilevel analyses including the individual, organizational and institutional factors [3, 19].

Our study addresses these understudied areas, for a country that has not been analyzed in this way yet: Switzerland. First, focusing on individual actors, we describe the extent of scientists’ engagement with non-scientific actors. We focus on politicians, journalists and industry representatives, i.e. three different important non-academic outside contacts [for an overview 3]. This is based on a census survey of Swiss professors, i.e. a specific but important subgroup of scientists. Second, we explain the extent of these contacts using statistical multi-level modelling that incorporates respondents’ individual attitudes and sociodemographics as well as their organizational and institutional contexts, using the comprehensive explanatory framework proposed by Perkman et al. [4, 19].

## 2. Describing and explaining scholars’ contacts with politicians, journalists and representatives of the economy: Reviewing the research field

A considerable number of studies has analyzed „the factors and processes that lead scientists to interface with nonscientists" [20]. Such studies, mostly based on standardized survey data, exist for various countries [for overviews 2, 4, 21] and disciplines [22–27], usually focusing on individual countries [e.g. 28–30] and single disciplines [e.g. 31, 32]. These studies provide insights into the degree of academic engagement and its antecedents.

### 2.1 Scholars’ contacts to journalists, politicians and industry representatives: Descriptive findings from prior scholarship

The respective studies show, firstly, that "a significant proportion of academics pursue academic engagement" [4]. Consulting for political and industry representatives, collaborative research or research for industry as well as contract research are comparatively common forms of academic engagement, with studies reporting, e.g., that more than half of UK [7, 33], Irish or Swedish scholars engage in such activities in their career [4]. D’Este and Perkmann [34] show that every second engineer and physical scientist in the UK was involved in collaborative or contract research or consulting within 24 months. 20% of German scholars publish together with industrial partners and 17% serve as paid consultants over a 12-months period [35]. Engagement with the news media or journalists is similarly high. Among 2.587 astronomers from different countries, 87% "reported engaging with the public […] frequently and regularly both through public events and the media" [31]. In stem cell research or epidemiology, 70% of scientists had at least one contact to journalists in a three-year time span, and every fifth had more than ten such contacts [36]. In climate research, more than two thirds had media contacts over the time of one year [32, 37]. In addition, many scholars take part in other public communication activities, such as writing articles in news media [38].

At the same time, research shows considerable differences between individual scholars, disciplines and countries [2, 4, 39, 40]. For example, the studies that have systematically compared scholars from different fields have shown that in Germany, more than 90% of scholars from communication sciences, history and philosophy have had at least 1 contact to journalists over the past 3 years, whereas this number shrinks to about 50% in mathematics, informatics and chemistry [39]. Similarly clear differences have been observed for exchanges with the industry in the UK [33] and politics in Austria [5, 6], for example. Furthermore, country differences have been observed. A comprehensive survey of astronomers shows country differences, with "higher activity among astronomers in South America and Africa [31].

### 2.2 Explaining academic engagement with politicians, journalists and industry representatives: Factors and findings from prior scholarship

Even though not all of the abovementioned studies attempted to *explain* academic engagement, the ones that do provide helpful insights for our analysis. They have taken many explanatory factors on different analytical levels into account, from scholars’ individual characteristics such as sex and age over organizational aspects like peer pressure to their embedding in different disciplines. Some studies have also included factors outside science–i.e. characteristics of the respective firms in industry relations [5], or networks in which scientific organizations may be embedded in [41]. However, these factors lay beyond our analytical focus. Scholars have used different combinations of these factors, and have not always tried to integrate them conceptually. Among the available conceptual models to explain academic engagement [e.g. 20, 42], the one proposed by Perkmann et al. [4] is arguably the broadest and most inclusive [for similar accounts 3, 43]. It distinguishes *individual factors* such as scholars’ sociodemographics and their attitudes towards academic engagement; *organizational contexts* such as peer and social norms or organizational characteristics like academic quality, organization incentives or working routines; as well as *institutional contexts* such as scholars’ disciplines or national contexts. These distinctions are useful to organize explanatory factors used in prior studies. The available scholarship shows that some of them are positively or negatively associated with academic engagement, while results remain ambivalent for others, indicating the need for more systematic research.

On the ***level of individual researchers***, findings about the role of *sociodemographic factors* are mixed. Sex seems to play a role, with men being more likely to interact with non-scientists–particularly with industry representatives [44, 45], but also with journalists [25, 27, 38], even though a few studies find no [31, 32] and even inverse effects [24]. Age differences were often included in the respective analyses, but with ambiguous results [4, 40]. A few studies also indicate that scholars’ nationality plays a role in their engagement, with foreign-born scholars being engaged less in their country of work than domestic colleagues [46].

Studies including the *status of individual scholars–*measured, e.g., via seniority [34], scientific productivity [47] and publications [31], or the occupation of leadership positions [32]–overwhelmingly show that status is an important factor driving academic engagement [3, 4, 39, 40]. This effect is likely grounded in the heightened standing of senior scholars which leads to them being approached more often [24], in the networks they have developed beyond academia [47] and in the organizational preferences for having senior scholars represent them [39].

The few studies that included whether scholars received a *communication training* [20, 31] or has skills for engagement activities more generally [3] demonstrate that this factor seems to increase outside contacts.

In addition, studies have shown that researchers’ *attitudes towards academic engagement* play a considerable role [3, 4, 40]. Researchers’ personal enjoyment influences their number of media interactions [48] and engagement more generally [26]. It has also shown that engagement is higher if scientists think it is their duty to inform the public and if they expect positive outcomes for themselves [31, 42, 49]. Likewise, scholars’ engagement-related "self-efficacy" [20] plays a role for the intensity of outside contacts [40, 42]. This reflects beliefs about whether scholars think they have the ability to interact with non-scientists based on their skills, and on their time resources [32, 48, 50]. Research has shown clear effects of researchers’ assessments of their own communication skills on public engagement [26, 49] and on the amount of media interactions [48]. In addition, the restricting effect of (perceived) time constraints on engagement activities has also been shown [49].

Findings about *scientists’ general use of news media and of online media*, which has been hypothesized to be connected to public engagement, have been mixed, with Dudo [20] showing small effects but Dunwoody et al. [48] showing none.

In addition to individual characteristics, ***organizational contexts*** have been taken into account [3, 4, 22, 31]. Scholarship suggests that relevant factors reside at this meso level [and that scholars themselves perceive organizational factors to be important, 39].

Findings about *social norms*–mostly operationalized as the (perceived) views of scientific peers [22] towards engagement [4, cf. 48, 49]–suggest that they are important moderators of academic engagement. (Perceived) Positive perceptions of engagement with industry representatives has been shown to increase the willingness of UK and German scientists to do so [47]. They also influence scholars’ engagement towards the news media [26, 42, 49, 51]. In addition, the anticipation of public reactions has been shown to influence the media interactions for the case of climate scientists [29]. But there are also some studies that find no such effects [20, 48].

Studies on the role of more general organizational characteristics–like them being a research university, privately or publicly funded etc.–have produced mixed findings [3]. Some studies show that organizational support or, conversely, organizational restrictions impinges upon academic engagement, albeit not strongly [20, 42], while others find no such effects [52]. In addition, the existence of bridging mechanisms or institutions–such as technology transfer mechanisms [4], stakeholder relations and PR offices–has been shown to have effects on the amount of individual scholars’ contacts to journalists [22] and on commercial enterprises [53, 54]. Generally, scholarship suggests that this organizational meso level is worth exploring further, given the still limited number of available studies on it and their ambiguous findings.

Apart from individual factors and organizational context, ***institutional contexts*** may influence academic engagement [3, 4]. Particularly relevant are *disciplinary affiliations*, whose role seems to vary depending on the type of engagement: While interactions with industry representatives are particularly common among scholars from applied fields such as engineering or computer science [5, 55–57], it has repeatedly been demonstrated that scholars from the humanities and social sciences have more interactions with the media and journalists [25, 40, 42].

### 2.3 Analyzing scholars’ contacts to journalists, politicians and industry representatives in Switzerland: Research questions

These studies are highly instructive, but in their entirety, they still have several gaps and shortcomings. First, prior scholarship has focused on a small number of select countries [2, 4, 58]. Second, the diversity of previous studies’ makes them difficult to compare. Partly, this is "a result of [their] different target groups, sampling strategies, and question designs" [39]. Partly, it is also due to the studies often analyzing specific disciplines–mostly natural sciences such as astronomy [31], epidemiology [36] or climate science [32]–, specific populations of scholars–like academy members [e.g. 42] or research council members [25], or specific kinds of engagement only–like interactions with industry [41] or contact to journalists [32]. In addition, third, more systematic research is needed to explain academic engagement, taking into account different factors on the levels or individuals, organizations and institutions [19, 38].

We aim to remedy some of these shortcomings in our study, focusing on Swiss professors’ contacts with journalists, politicians and industry representatives, using a systematic census analysis across all scholarly fields, and by coupling the descriptive findings with an explanatory analysis guided by a comprehensive model that includes individual, organizational and institutional factors [following 4].

In doing so, we focus on a case that is interesting yet under-researched: Switzerland. The country hosts some of the leading universities in the world, like the Swiss Federal Institutes of Technology in Zurich and Lausanne. Half of its 12 universities rank in the top 200 universities globally, according to several international rankings [59]. It higher education sector has grown strongly, and many institutions are oriented strategically and politically towards Swiss society and particularly partnerships with the industry [60, 61]. So far, however, little is known about academic engagement in Switzerland [for one of the few exceptions see 62].

This translates into two research questions: RQ1: *How often do professors engage with politicians*, *journalists and industry representatives*? and RQ2: *How can the extent of this engagement be explained with factors on the individual*, *organizational and institutional level*?

## 3. Data and methods

### 3.1 Sample

We were able to access the proff.ch database which was provided until 2016 by CRUS (Conférence des Recteurs des Universités Suisses), the umbrella organization of Swiss universities. It includes all professors employed at Swiss universities, with information on employment status, field of work, and contact details. Initial checks showed the database was reliable: No professor employed at a Swiss university at the time was missing. Only the status of a few professors who had retired or changed to foreign universities was not updated, so we excluded them manually from analysis. While the Swiss system has a different structure than the US system, our sample can be compared to a sample covering all faculty positions in the US context (assistant to full professors).

Data for our study was collected via standardized online survey. The basic population of the survey were all professors working at Swiss universities. The proff.ch database identified 5,859 professors, whom we sent the link to an online questionnaire as well as two reminders in May 2017. Respondents could choose between German, French, and English versions of the questionnaire. 1,151 professors completed it (20% response rate) and 1,058 of those indicated a Swiss university as their current affiliation and selected a discipline. For our statistical models we had to drop an additional 27 cases as they work for special research institutions (see S2 Appendix) for which no organizational variables are available. Furthermore, for each model we only included cases that have no missing data for any of our three outcome variables. We also had some single missing values for our predictors for which we used all other predictors to do data imputation as we could not identify a specific bias with regard to the missing variables [63]. As recommended in the literature we created 20 imputed data sets that were all used together in our models [64]. Almost all missing values could be imputed which allowed us to add to our models (journalist n = 1022, politicians n = 1019, industry = 1016).

Compared to the statistical description of Swiss professors provided by the Swiss Federal Statistical Office [65], our sample seems representative. Regarding gender, disciplines and university affiliations, there are no major deviations from Swiss professors in general in our sample (see the table in the S1 Appendix). In the sample, 24% of respondents are female (n = 254), compared to 22.2% (n = 969) in the population. Also, disciplines are well distributed in the sample. To compare the distribution of the more granular disciplines in our survey with the only six disciplines provided by the Federal Statistical Office we combined the sub-disciplines to the main disciplines mentioned in the data provided by the government [65]. It is evident that our sample corresponds quite well with the basic population based on the broader discipline categories. The strongest deviation can be found in a slight overrepresentation of the natural sciences, which make up 28.92% (n = 306) of professors in the sample, compared to 24.25% (n = 1058) in the population. This difference may stem from the different taxonomies as Engineering and Technology are slightly underrepresented in the sample (6.05% compared to 9.56%), for instance. Furthermore, the distribution of Universities in the sample corresponds well with the total population. Only the two federal technical universities in Lausanne (EPFL) and Zurich (ETH) are slightly underrepresented, while the University of Zurich is slightly overrepresented (see supplementary files).

### 3.2 Measurements

Three outcome variables were measured with a survey question adapted from previous studies [27, 32, 66]: Respondents were asked how many times (they could select numbers between 0 and 100) they had professional contacts with journalists (*m* = 5.62, *SD* = 9.97), politicians (*m* = 2.41, *SD* = 4.95) or industry representatives (*m* = 5.42, *SD* = 10.92) in the past 12 months. With this broad question, we measure potential engagement as described by Perkman et al. [4] and across different fields, but do not differentiate between formal and informal activities or differentiate individual activities [like, e.g., 33].

To measure the described explanatory factors, variables were derived from previous studies and partly adapted for our purposes. Most of them were measured by 5-point rating scales with labeled anchor points (1: strongly disagree; 5: strongly agree–see S1 Appendix for an overview of all used questions and items). We calculated mean indices for variables such as internal efficacy or attitudes. We included one mode 2 item as well as the "duty to inform the public"-item as single items as they did not yield acceptable reliability scores as an index. The number of publications were normalized by discipline (divided by the maximum value in the discipline–thus a number between 0 and 1). We also included variables on the institutional level such as the number of press releases (counting the number of annual releases by directly contacting each institution) and the third party funding-ratio [based on government statistics, 22]. We additionally added on university level the internal ranking of Swiss universities based on the Times Higher Education ranking. Table 1 gives an overview over all variables used in our analysis (see the S1 Appendix for all the questions and items).

**Table 1:** Overview of all predictors and outcome variables.

Variable Name	Description [incl. reliability]	Mean	SD	n
***Attitudes activity***				
mode 2 practitioners	"Scientists should work together with practitioners outside of science." (1: strongly disagree; 5: strongly agree)	3.68	1.07	1019
duty to inform public	"Scientists have a duty to inform the public about their research." (1: strongly disagree; 5: strongly agree)	4.22	0.94	1028
attitude activity	Enjoyment in engaging in different activities, i.e."Explaining my research and its results to the public." (1: strongly disagree; 5: strongly agree), 4 items, α = 0.77	3.80	0.91	1019
***Social norms***				
negative extrinsic rewards	Concerns, i. e. "I may receive critical reactions from peers." (1: strongly disagree; 5: strongly agree), 2 items, α = .72	2.09	0.92	1019
***Communication self-efficacy and constraints***				
communication self-efficacy	Perceived ability to interact with non-scientists, i.e. their skills. (1: strongly disagree; 5: strongly agree), 4 items, α = .81	3.33	0.83	1018
reservations time	"I lose valuable research time." (1: strongly disagree; 5: strongly agree)	2.87	1.29	1024
***Status of scholar***				
research experience	Number of years in research (count)	23.43	8.13	1029
publications normalized	Number of publications ("So far in your career, how many scientific publications have you authored or coauthored approximately?"), count normalized by discipline (divided by maximum number).	0.27	0.22	1027
management position (yes)	"Do you currently occupy a management position in your institution (e. g. department head, dean, director)?" 1 = yes, 0 = no	42%		1019
***Sociodemographic factors***				
nationality (Swiss)	Where were you born? (categorical) Created a dichotomous variable; 1 = Swiss, 0 = not Swiss	47%		1031
sex (male)	Dichotomous variable: 1 = male, 0 = female	76%		1025
birth year	Year born	1964	8.16	1020
***Communication or media training***				
Communication training	Have you ever had formal training in public communication or media skills? 1 = yes, 0 = no	28%		1020
***Scientists general use of news media and of online media***				
traditional media consumption	„How closely do you follow the media […]?" (1: Not closely at all; 5: Very closely), 2 items, α = 0.73	3.78	0.84	1022
social media consumption	"How closely do you follow […] social media?" (1: Not closely at all; 5: Very closely), 2 items, α = 0.93	1.81	1.08	1030
***Disciplinary affiliations***				
discipline	"Which of these disciplines describes your field of research the closest?" (categorical–OECD taxonomy)			1031
***Organizational influences***				
number of press releases (university)	The number of press releases in 2016 (count)	62.57	33.11	1031
third party funding-ratio (university)	Ratio between total budget and third party funding (based on statistics from the Federal office of Statistics)	0.36	0.09	1031
autonomy (yes)	"Do you have to seek approval from your institution before talking to a journalist?" 1 = no, 0 = yes	80%		1014
support media relations	"I inform the media relations department of my university about interesting research I conduct" (1: strongly disagree; 5: strongly agree)	2.97	1.39	1023
university	"At which Swiss university do you work?" (categorical)			1031
university ranking (university)	Ranking of Swiss universities based on the Times Higher Education Ranking in 2017.	4.89	2.41	1031
***Outcome variables***				
contacts with	"Please estimate: How many times, in the past 12 months, have you had professional contact with the following types of actors?" (count– 0–100)			
Journalists median 3				
		5.62	9.97	1026
Politicians median 1		2.41	4.95	1019
		5.42	10.92	1017
Industry median 2				

### 3.3 Models

Because our outcome variables consist of count data (measuring the number of contacts in the last 12 months) and are slightly overdispersed (*SD* is higher than the mean for all three outcome variables), we calculated negative binomial regression models with random intercepts for disciplines and universities. In doing so, we account for the fact that scientists from specific disciplines would not have equally many contacts with journalists, politicians and industry representatives absent all of the predictors in our model [39].

We calculated Bayesian models with R, using Stan [67] within the brms package. We rely on a Bayesian approach, firstly, because it performs better than frequentist regression models when data has a multilevel structure [68]. Secondly, frequentist p-values and confidence intervals are often misinterpreted [69]. The Bayesian approach solves this problem as the parameter estimates and the „credible intervals support an interpretation of probability in terms of plausibility" [70]. Thus, we can directly interpret the credible interval in a Bayesian model and claim that there is 95% probability that the true value is in the interval. From a more practical perspective, models with complicated multilevel structure that do not converge with a frequentist approach usually converge without problem with a Bayesian approach.

In order to calculate Bayesian models, we need to define so-called priors for all estimates. Even though we have some expectations about our estimates based on prior research, we only used weakly informative priors. We, for example, assume our estimates to be not exorbitantly large, but besides that we do not have specific knowledge that justifies more specific priors. This means we use very conservative priors which still leads to more robust estimates.

We centered and scaled all of our continuous predictor variables. For each of the three outcome variables, we ran a separate model. Using four sampling chains for each of our 20 imputed data sets, we drew 1,000 warmup iterations followed by 4,000 sampling iterations for each chain. The results are pooled across models estimates [64]. All estimates reached an R-hat [scale reduction factors; 71] lower than 1.05. All models converged and the traceplots show well mixing and stationary chains for all estimates.

## 4. Results

### 4.1 Descriptive results

**Fig 1:**
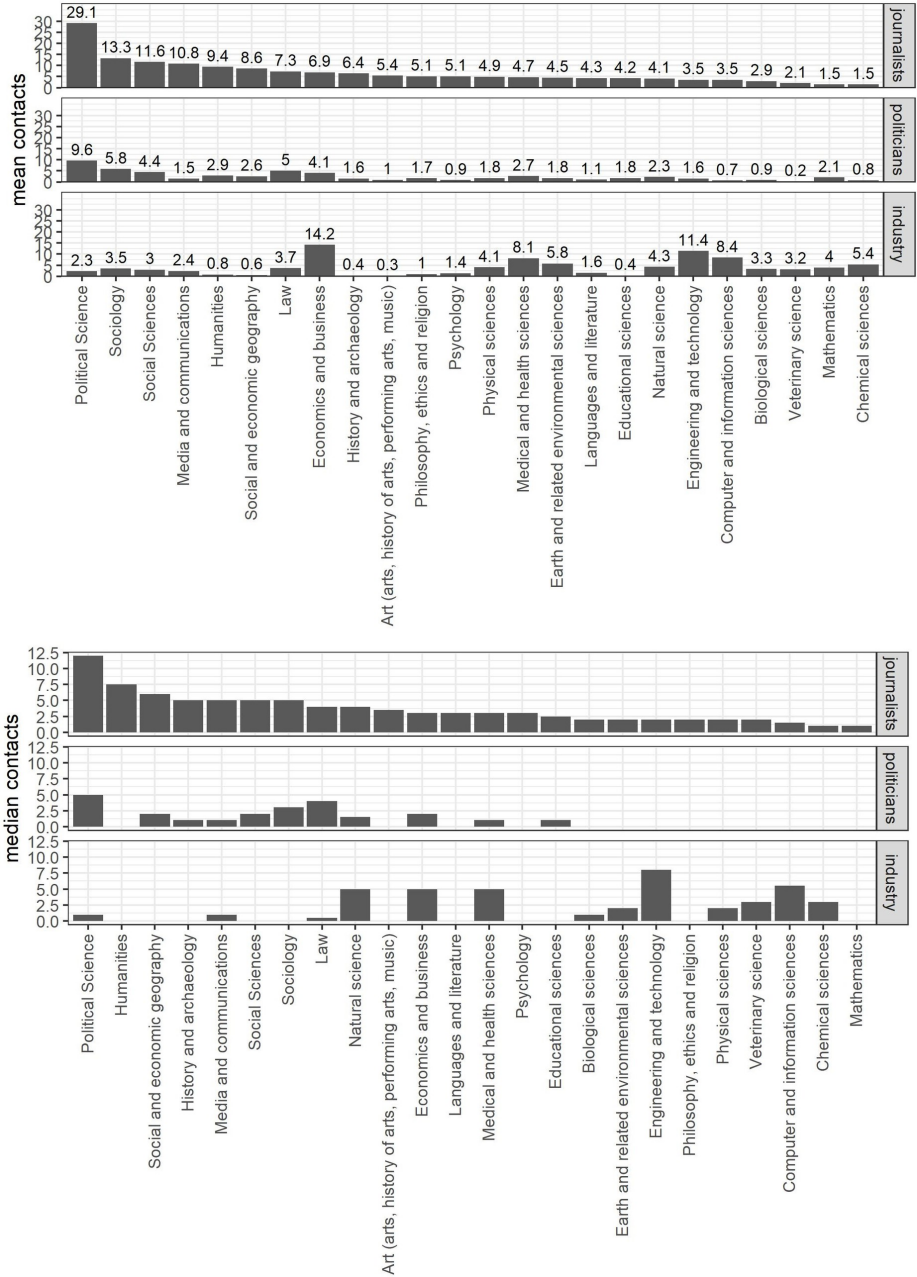
Description of scientists’ contacts to politicians, journalists and industry representatives. Depicted are the average no. of professional contacts the surveyed scholars have to journalists, politicians and industry representatives annually. The upper part shows the means; in the lower part the medians are visualized for every discipline.

On average, the surveyed professors have more than one professional contact per month with either politicians, journalists or industry representatives: Respondents report 13.3 annual contacts across all three groups of stakeholders (SD = 18.59; see Fig 1). Most of these contacts are to journalists, to whom the surveyed scientists have 5.62 annual professional contacts (SD = 9.97) year on average. Contacts to industry representatives occur almost equally often, i.e. 5.42 times per year (SD = 10.9). Contacts to politicians are less common. Respondents average 2.41 professional contacts with this stakeholder group per year (SD = 4.95).

The relatively high standard deviations across these contacts indicate, however, that frequencies of external contacts are unevenly spread across respondents. Around 10% of the respondents (n = 117) have 50% of all contacts to journalists. However, around 84% (n = 866) of respondents had at least one contact with a journalist. Furthermore, while 50% had at least one contact with a politician, 7.4% (n = 75) have 50% of all contacts to politicians. 64% of the respondents had at least one contact with industry representatives and 8.6% (n = 87) of the respondents have 50% of all contact to the industry representatives.

In addition to these individual differences, the overall means mask clear differences between scientists from different disciplines. On average, professors from the social and economic sciences have most external contacts. Political scientists report an average of more than 40 such contacts per annum, economists 28.7, sociologists 22.5 and professors who indicated to work in the social sciences more generally 19.3. In turn, professors from certain disciplines in the arts as well as veterinary scientists have the least amount of such contacts, with annual means ranging between 5.3 and 6.9, respectively.

In addition to the different number of outside contacts, the disciplines also vary in the nature of these contacts: Social scientists have a lot more professional interactions with journalists and politicians compared to other disciplines, particularly political scientists (28.9 and 9.2) and sociologists (13.0 and 5.8). In contrast, social scientists do not interact often with industry representatives. Contacts to representatives from corporations etc. are mostly found among economists (14.0) and professors from engineering and technological sciences (11.6). Natural scientists from fields such as physics, chemistry or biology have fewer outside contacts than other professors on average, and if they do, those are mostly to journalists and industry representatives. Professors from the arts often have few contacts to politics and industry. The, albeit relatively few, outside contacts they have are mostly to journalists.

### 4.2 Explanatory results

We calculated negative binomial regression models with random intercepts (for disciplines and universities each) for contacts to journalists, politicians and industry representatives separately (see Table 2).

**Table 2:** Estimates of all three models (Bayesian negative-binomial regressions with varying intercepts for universities and disciplines).

	journalists			politicians			industry representatives		
variable	estimate	2.50%	97.50%	estimate	2.50%	97.50%	estimate	2.50%	97.50%
mode 2 practitioners	0.03	-0.04	0.10	0.18*	0.07	0.29	0.19*	0.09	0.30
duty to inform public	0.11*	0.04	0.19	0.07	-0.05	0.18	-0.05	-0.15	0.05
attitude activity	0.22*	0.14	0.29	0.34*	0.22	0.46	0.04	-0.06	0.14
negative extrinsic rewards	-0.03	-0.10	0.04	-0.03	-0.14	0.08	0.00	-0.10	0.10
communication self-efficacy	0.25*	0.18	0.31	0.15*	0.04	0.25	0.13*	0.04	0.22
reservations time	-0.08*	-0.15	-0.01	-0.17*	-0.28	-0.06	-0.10	-0.20	0.00
Birth year	-0.04	-0.16	0.07	-0.16	-0.35	0.02	0.05	-0.11	0.21
research experience	-0.07	-0.18	0.04	0.07	-0.10	0.24	-0.04	-0.19	0.11
publications normalized	0.19*	0.11	0.27	0.16*	0.04	0.28	0.16*	0.04	0.27
sex (male)	0.07	-0.09	0.23	-0.07	-0.34	0.19	0.50*	0.27	0.74
nationality (Swiss)	0.22*	0.09	0.36	0.39*	0.17	0.61	-0.09	-0.29	0.11
social media consumption	0.12*	0.06	0.19	0.00	-0.10	0.11	-0.02	-0.12	0.08
traditional media consumption	0.02	-0.05	0.09	0.07	-0.04	0.18	0.14*	0.04	0.24
autonomy (yes)	0.27*	0.07	0.46	-0.19	-0.47	0.10	-0.53	-0.77	0.29
management position (yes)	0.28*	0.14	0.41	0.55*	0.34	0.76	0.36*	0.17	0.56
third party funding-ratio	-0.06	-0.16	0.08	-0.03	-0.25	0.22	0.10	-0.14	0.33
number of press releases	0.04	-0.08	0.17	0.04	-0.23	0.27	0.16	-0.07	0.43
University ranking	0.09	-0.01	0.19	-0.01	-0.20	0.19	-0.17	-0.35	0.02
Communication training	-0.14	-0.29	0.01	-0.30*	-0.54	-0.07	-0.07	-0.29	0.14
support media relations	0.14*	0.07	0.21	0.20*	0.09	0.31	0.05	-0.05	0.14
intercept	1.07	0.73	1.41	0.42	-0.12	0.96	0.90	0.30	1.50
Bayes-R	0.33			0.43			0.28		
Complete cases	1022			1019			1016		

Models with the number of contacts with journalists, politicians, and industry representatives as the dependent variables. Note: The range between the 2.5% and 97.5% quantiles is the so-called 95% credible interval (CI). Given the data, there is a 95% probability that the parameter of interest lies in this range. We calculated the Bayes-R for each model. We used standardized values for all non-binary predictors.

* indicates if 0 is not in the 95% interval.

The first model tested the relevance of these predictors for *scientists’ contacts to journalists*. We calculated the Bayes-R according to Gelman et al.’s [72] recommendation. The model yielded an explained variance of 0.33. The analysis shows, first, the strong relevance of attitudinal variables: Respondents’ self-efficacy–i.e. their confidence in their ability to interact with journalists, explain their topic and findings well etc.–increases the number of contacts to journalists (there is a 95% probability that the true value lies between 0.18 and 0.31). Also, scientists’ attitude towards communication activities indicates a positive relationship with the number of journalist contacts. Second, the professors’ media use patterns are important: An intense social media use (other than an intense consumption of traditional news media) increases the number of contacts to journalists. Third, respondents’ status is linked to journalist contacts: The more productive a professor has been scientifically–i.e. the more publications s/he has published compared to colleagues from the same field–the more contacts s/he has with journalists. Also, scientists who occupy management positions in their organizations have more contacts to journalists. In addition to these micro-level factors, one organizational variable is a strong predictor: Professors who have the autonomy to decide themselves whether and how often they want to engage with journalists have more such contacts than colleagues who need organizational approval to engage with journalists. Also, professors that are actively trying to provide the media relations department at their university with information have more contacts with journalists.

Interestingly, the model explaining the number of *scientists’ contacts with politicians–*which yields an even better explanatory power (Bayes-R 0.43)–shows similar results. This is in line with the descriptive findings that showed that professors who often have contacts with journalists also often have contacts with politicians. Coefficients between the different types of contact are .58 for contacts with journalists and contacts with politicians, .09 for contacts with journalists and contacts with industry representatives, and .26 for contacts with politicians and contacts with industry representatives. For an explanation of contacts with politicians, the respondents’ self-efficacy, their attitude towards outside communication activities, their number of publications, no reservations about losing valuable time for research, and the occupation of management positions are all strong predictors. In addition, and again similar to journalist contacts, respondents’ nationality is an important predictor. Additionally, if professors believe that they should cooperate with practitioners, they have more contacts with politicians. Again, the media relations department is also an important predictor.

In contrast to the first two, the model explaining *scientists’ contacts to industry*–with a considerably lower explanatory power of 0.28 (Bayes-R)–shows different results. Self-efficacy, the attitude towards communication activities, the number of publications as well as the occupation of a management position all show substantial positive relationships with the outcome variable. In addition, this is the only model in which the gender of a professor is relevant: Male professors have more contacts with industry representatives. Instead of the social media variable, which seems to be a relevant variable for the number of contacts with journalists, the traditional news media consumption has a positive effect on the number of contacts with industry representatives. Nationality, however, is not a relevant predictor.

When comparing these results across the three models, it becomes evident that the explanatory models for professors’ contacts to politicians, journalists and industry representatives have some parallels: Self-efficacy, attitudes towards communication activities, respondents’ publications numbers and management positions are equally strong positive predictors (as they are all scaled) in all three models.

**Fig 2:**
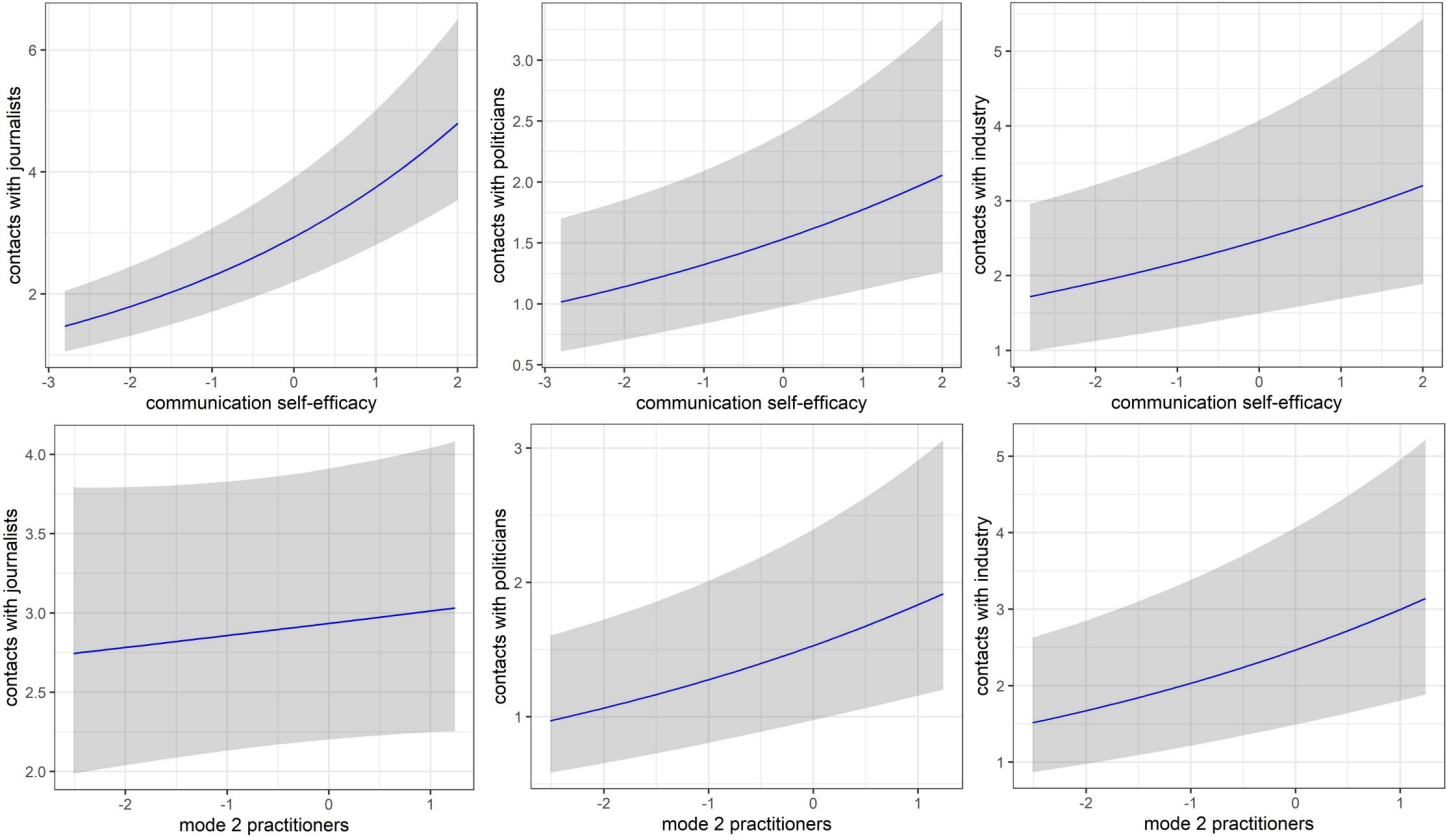
**Marginal effects for the predictors internal efficacy (top) mode 2 practitioners (bottom) (M = 0, SD = 1) for all three outcome variables.** Gray area shows the credible intervals (95%).

It is notable that self-efficacy is a better predictor for the number of contacts with politicians and journalists than for the number of contacts with industry representatives. This is clearly visible when the marginal effects are visualized for every level of the internal efficacy while keeping all other predictors constant (see Fig 2). The internal efficacy has clearly the narrowest credible intervals for the number of contacts with journalists.

One other variable is noteworthy: The mode 2 practitioner variable shows a positive relationship for the number of contacts with politicians and industry representatives. The marginal effects visualizations clearly show that this variable does not predict the number of contacts with journalists well with an almost horizontal line and extremely wide credible intervals (see Fig 2).

Interesting is also the connection between news media consumption and contacts with industry representatives. Social media use is only a substantial predictor for the number of contacts with journalists. The nationality only plays a potential role for the number of contacts with journalists as well as the number of contacts with politicians.

**Fig 3:**
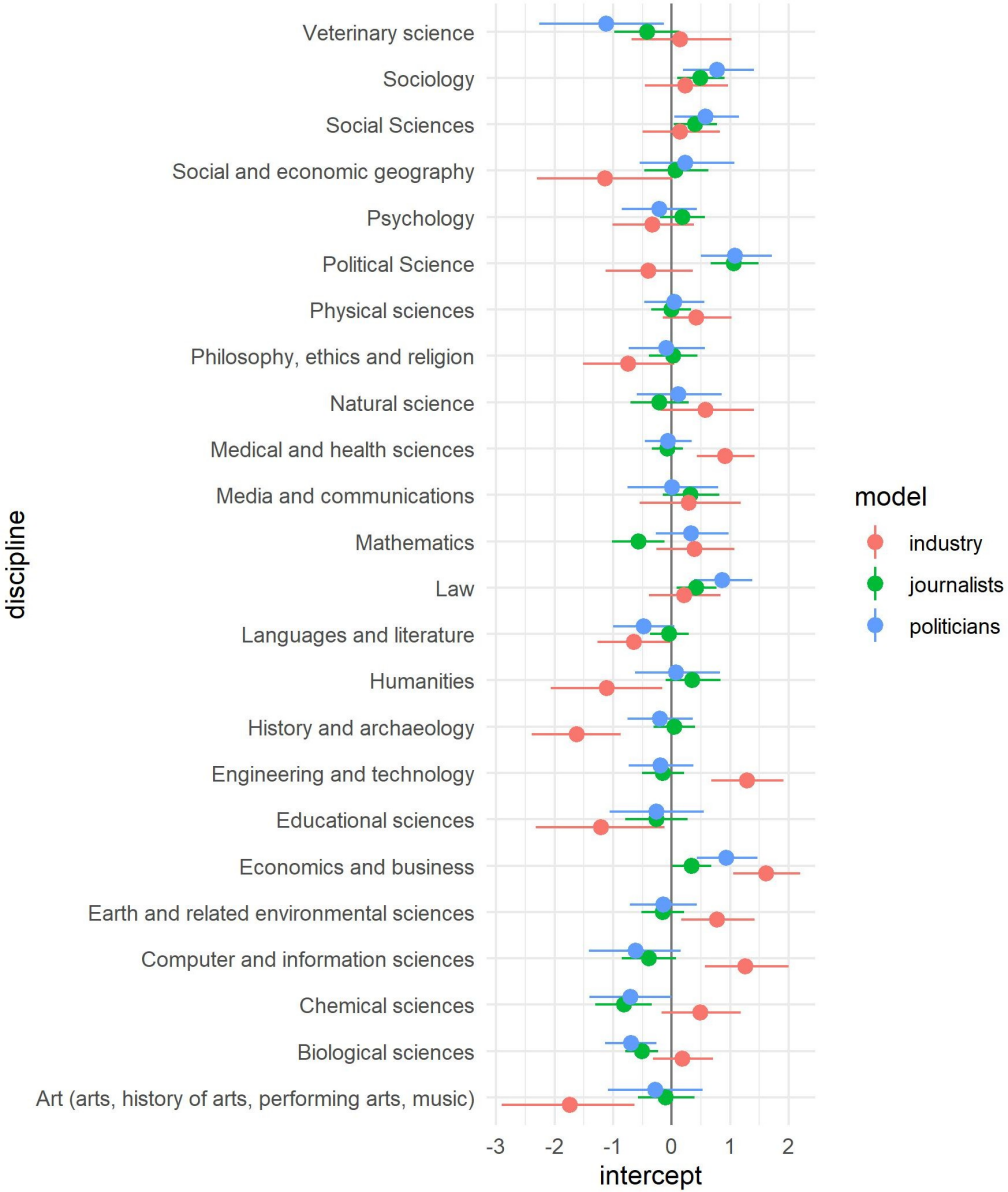
Varying intercepts for each discipline for all 3 models. Lines show the 95% CI of the intercepts.

With regard to the institutional variables we could not find any substantial effect. All three models clearly show that the disciplines as varying level two intercepts clearly explain some of the variance whereas the level two institutional variables as well as the varying intercepts of the universities (CI of all varying intercepts include 0) show no substantial influence. However, for the disciplines the results observed in Fig 1 are present in Fig 3 with the varying intercepts. Professors from disciplines like computer sciences, economics and business, medical sciences or engineering have a higher probability to have contacts with industry representatives. On the other hand, sociology, political science or law have a higher probability to have contacts with politicians and journalists.

## 5. Discussion

Using comprehensive census survey data on professors from all Swiss universities, we analyzed how often they have professional contact with politicians, journalists and industry representatives. In a second step, we aimed to explain the amount of these contacts, including explanatory factors on the individual as well as organizational and institutional levels.

On the one hand, and in line with previous scholarship [2], our study showed that academic engagement is quite common [2, 39]. Many Swiss professors are in contact with journalists, politicians and industry representatives. Across all fields and all kinds of contacts, respondents average slightly more than one such contact per month. Most of these contacts are with journalists–which have been shown to be quite common before, particularly in publicly discussed fields like the biosciences or climate science [37, 39]–and industry representatives. Contacts to politicians are less common [3].

However, there are pronounced individual and disciplinary differences, similar to other countries [2, 4]. Overall, Swiss social scientists and economists have the largest amount of external contacts, whereas arts professors as well as veterinary professors have the least. In addition, and also in line with prior findings [e.g. 33], the different disciplines have different kinds of outside contacts: Social scientists have most interactions with journalists and politicians–but not with industry representatives, who mostly interact with economists and professors from engineering and technological sciences [cf. 3]. Our descriptive results with regard to contacts with journalists are similar to the patterns identified in the German context [45]. However, while in our study the disciplines with most professors without any contact (Mathematics 40%; Chemical Sciences 36%; Computer and Information Science 35%) are the same as in Germany, more respondents had at least once a contact with a journalist, probably due to our focus on professors and not, like earlier studies, on early career researchers [e.g. 32].

Our explanatory models show that several variables are important predictors for all three types of outside contacts. Among these predictors, individual level ones are the most impactful [similar to 28]: First, the higher respondents’ self-perceived internal efficacy, the more contacts they have with outside actors. This attitudinal measure was a strong predictor of outside contacts with all three external groups. This is consistent with a large amount of previous research [26, 32, 49, 50]. It is noteworthy that all aspects included in this measure–being able to explain one’s research topics, feeling comfortable in front of a microphone etc.–can be improved by training. Even though scientists from different disciplines may not equally willing to take part in science communication training [73], their self-assessed efficacy towards handling outside contacts could be improved and, thus, these contacts could be intensified. Overall, communication training does not play an important role as predictor. With regard to the underlying mechanism of this relationship, it is possible that our result is the effect of a reinforcing mechanism: Through contacts with outside actors, professors increase their skills, leading to even more outside contacts. Future studies using longitudinal research designs could investigate this relationship further as there might be a problem with endogeneity especially for professors with a high number of contacts each year.

In contrast, organizational variables (third-party funding ratio, number of press releases, university ranking) did not explain the number of outside contacts. In the context of our study it can be concluded that individual-level factors as well as the disciplinary background of a professor are more important.

However, as there is a difference between the number of contacts between universities, future studies should try to identify further organizational variables that might explain the differences. Such studies could take further organizational factors into account, like the proximity to political bodies, corporations or media organizations [cf. 74], the organizational networks in which universities are embedded [41] or the characteristics of the political, industry or media organizations that scientists may actually interact with [5].

In our study, disciplinary affiliation seems to have a larger effect on engagement than organizational factors if the varying intercepts in our models are analyzed. Our multilevel models allowed us to clearly test whether overall the organizational, individual or disciplinary factors have an effect on the number of contacts. While some of the disciplines with a higher probability to lead to engagement are more applied than the other disciplines (e.g., computer sciences or medical sciences), we checked whether on discipline level our variable measuring the attitude towards work with practitioners shows the same rank order as the rank order for the contacts and could not find a significant relationship (Spearman’s rho all not significant).

Second, a strong predictor for the number of contacts with journalists and also a potential factor for the number of politicians and industry representatives is the number of publications of a given professor. This finding is also in line with previous research [19]. There are two possible mechanisms underlying this relationship. First of all, it is possible that the more a professor publishes, the higher his or her visibility will be–consistent with findings that a higher scientific status and seniority increase the number of scientists’ outside interactions [24, 25, 48]. It is possible that the number of publications is a proxy for the general activity of a professor: professor who are active academically also try to communicate more with outside actors.

Third, scientists who occupy management positions in their organizations have more contacts with all three outside actors. Again, this finding corresponds to previous research which has documented that scientists in management positions have more outside contacts, are more often contacted by external stakeholders and also feel more confident representing their organization [24, 31, 32, 51].

Scientists’ social media use only predicts contacts with journalists. In Switzerland as well as in many other countries Twitter is a popular platform amongst journalists [75, 76]. It could be possible that professors who are more active, especially on Twitter, have a stronger visibility and receive potentially more requests from journalists. As we only asked about contacts, not the context in which these contacts took place, it would be even possible that professors had their contacts with journalists over social media platforms. Future surveys should further investigate this relationship and ask more specifically about the context in which these contacts took place and differentiate between formal and informal contacts [4]. It is also noteworthy, that strength of collaboration with media relation departments predicts the number of contacts with politicians and journalists.

Gender differences–which were found in a previous study analyzing outreach and engagement activities of scholars of a Swiss university [27]–were not pronounced in our findings. They do not significantly influence the number of professors’ contacts to journalists or politicians, but partly explain contacts with industry representatives. This may mirror a lack of recognition of female professors [the so-called “Matilda Effect”, 27] which may be particularly pronounced among (Swiss) corporations, where gender equality is less developed compared to the Swiss political system [77] or Swiss journalism [78].

Lastly, a cultural predictor proved to be relevant in two explanatory models: The nationality of the surveyed professors positively predicted the number of their contacts with journalists and politicians. Being Swiss leads to more contacts, especially to politicians. This finding could be related to language–if a professor does not speak any of the Swiss national languages well enough, s/he might find it difficult to communicate with domestic stakeholders. It could also be connected to the cultural familiarity and national identity of the respondents–foreign-born professors may know and identify less with Swiss matters and, thus, engage themselves less in them. It could also be a matter of pre-existing networks, with Swiss-born professors having stronger personal networks in Switzerland. It is notable, however, that the professors’ nationality does not predict the number of contacts with industry representatives, which might be explained with the specific research focus of more technical disciplines in which the national context or cultural aspects matter less.

While the study at hand extends prior scholarship in meaningful ways–mainly because it presents an analysis of different outside contacts, analyzes scientists across disciplines, and provides explanatory models integrating individual and organizational variables in a multilevel structure–it is limited in other ways: First, aiming to capture academic engagement across different fields, we assessed it with a generic measure, focusing on individual scientists only and focusing on their ‘professional contacts’ to the most relevant stakeholder groups. Prior scholarship has shown that a broad variety of diverse ways of engagement exists, from news media appearances, public lectures and school visits [e.g. 40] over working in the corporate sector part-time or engaging in public-private partnerships [e.g. 2] to political consulting [e.g. 79], that also reside on individual and organizational levels [e.g. 5, 6]. We focused only on one part of that here.

Second, we only surveyed professors, who differ from other academic staff in their experience, visibility and job security, among other factors. And even though the sample does not seem to deviate strongly from the basic population of Swiss professors, we could only check this for the few dimensions where data on all Swiss professors are available from the country’s Federal Statistical Office. Furthermore, respondents were self-selected and reported their outside contacts themselves. We were not in the position to crosscheck these claims with the actual contacts of the respondents with, for example, politicians. In addition, we have no idea about the nature, content and quality of those interactions. Compared to studies with samples covering different kinds of researchers on all hierarchical levels [e.g., 45], our results appear slightly weaker.

Third, we limited our study to contacts to politicians, journalists and industry representatives, as these groups have been interpreted theoretically as the most relevant outside stakeholders [9, 15]. But similar studies for contacts with other societal stakeholders–such as representatives of churches, of civil society, or artists–would be equally needed. Furthermore, the three societal-stakeholders could be analyzed in more detail in future studies as we only used rather broad categories.

Fourth, the quality of the explanatory models presented here should be improved in future studies in, at least, two ways: Specific explanatory models for scientists’ contacts to politicians and industry representatives are needed, and longitudinal explanations would be helpful to distinguish correlational relations between certain concepts from causal linkages as there could be a problem with endogeneity with regard to professors with regular engagement activity.

## Supporting information

S1 Appendix (DOCX)

S2 Appendix (DOCX)
