# Supplementary material for: Beyond the ivory tower: Measuring and explaining academic engagement with journalists, politicians and industry representatives among Swiss professors
Source: PLoS One. 2021 May 21;16(5):e0251051. doi: 10.1371/journal.pone.0251051 (PMC8139464; doi:10.1371/journal.pone.0251051)
Supplement: S1 Appendix — (DOCX) [file pone.0251051.s001.docx]

**Email invitation**

The media reports on scientific topics on a daily basis. But how do Swiss professors see media coverage and what are their experiences with the media? The Chair for Science Communication at the University of Zurich is currently conducting a survey on these topics. We would highly appreciate your participation!

**Questions used in this study**

# *Opinions vary about the relationship between science and the public.* Please indicate for the following statements the extent to which you agree or disagree.

1

strongly disagree

5

strongly agree

24

3214

414

| Scholars have a duty to inform the public about their research.  (*duty to inform public*) |
| --- |
| Scientists with should work together with practitioners outside  of science.  (*mode 2 practitioners*) |

# How closely do you follow the media and social media?

1
Very

closely

5
Not closely at all

414

3214

24

| Media coverage in general (*traditional media consumption*) |
| --- |
| Media coverage about science (*traditional media consumption*) |
| Social media communication in general (e. g. Facebook, Twitter, blogs) (*social media consumption*) |
| Social media communication about science (e. g. Facebook, Twitter, blogs) (*social media consumption*) |

# Skills necessary for public communication may differ from skills required to do research and communicate it to peers. Do you personally find the following activities difficult or easy? (*communication self-efficacy – 4 items*)

414

3214

24

1

Very difficult

5

Very easy

| Explain scientific facts in a way that lay people can understand |
| --- |
| Adjust to different kinds of lay audiences |
| Get my research published in the media |
| Write in a way that a media audience can understand |

# Please indicate for each of the following activities how much you personally would enjoy engaging in this activity. (*attitude activity – 4 items*)

5
Enjoy very much

1

Do not enjoy at all

414

3214

24

| Explaining my research and its results to the public |
| --- |
| Describing possible practical uses of my research |
| Evaluating political decisions based on my professional expertise |
| Contributing to public debate about policy related to science |

# Have you ever had formal training in public communication or media skills? (*Communication training*)

| Yes  No  |
| --- |

# To what extent do the following concerns keep you personally from media contact?

5

strongly agree

1

strongly disagree

3214

24

414

| I lose valuable research time (*reservations time*) |
| --- |
| I get critical reactions from peers (*negative extrinsic rewards*) |
| I get critical reactions from the public (*negative extrinsic rewards*) |

# Please estimate: how many times, in the past 12 months, have you had professional contact with the following types of actors? (*dependent variables*)

| Journalists ___ times |
| --- |
| Politicians ___ times |
| Industry representatives ___ times |

# Do you have to seek approval from your institution before talking to a journalist or other member of the media? (*autonomy*)

| Yes  No  |
| --- |

# To what extent do you agree with the following statement?

5

strongly

agree

1

strongly
disagree

24

3214

414

| I inform the media relations department of my university about any  interesting research I conduct. (*support media relations*) |
| --- |

# What is your gender? (*sex*)

| male  female  |
| --- |

# What year were you born? (*birth year*) ___

# Which of these disciplines describes your field of research best? (*discipline*)

Drop-down list with disciplines (OECD)

# What is your nationality? (*nationality*)

# How many years have you been in research? (*research experience*) ___

# Besides your professorship: do you currently occupy a management position in your institution (e. g. department head, dean, director)? (*management position*)

**Yes** **No**

# So far in your career, how many publications have you authored or coauthored? (*publications normalized*)

|  |
| --- |
