# Supplementary material for: Beyond the ivory tower: Measuring and explaining academic engagement with journalists, politicians and industry representatives among Swiss professors
Source: PLoS One. 2021 May 21;16(5):e0251051. doi: 10.1371/journal.pone.0251051 (PMC8139464; doi:10.1371/journal.pone.0251051)
Supplement: S2 Appendix — (DOCX) [file pone.0251051.s002.docx]

| **Institution** | **n Sample** | **% Sample** | **n BfS** | **% BfS** |
| --- | --- | --- | --- | --- |
| Universität Zürich | 214 | 20.23% | 647 | 14.80% |
| Université de Genève | 158 | 14.93% | 640 | 14.70% |
| Université de Lausanne | 136 | 12.85% | 591 | 13.50% |
| Universität Bern | 132 | 12.48% | 534 | 12.20% |
| Universität Basel | 112 | 10.59% | 391 | 9.00% |
| Eidgenössische Technische Hochschule Zürich | 80 | 7.56% | 489 | 11.20% |
| Ecole polytechnique fédérale de Lausanne | 51 | 4.82% | 335 | 7.70% |
| Université de Fribourg | 51 | 4.82% | 254 | 5.80% |
| Université de Neuchâtel | 38 | 3.59% | 127 | 2.90% |
| Universität St. Gallen | 29 | 2.74% | 175 | 4.00% |
| Università della Svizzera italiana | 15 | 1.42% | 103 | 2.40% |
| Universität Luzern | 15 | 1.42% | 73 | 1.70% |
| IHEID Geneva* | 12 | 1.13% | NA |  |
| Paul Scherrer Institut* | 7 | 0.66% | NA |  |
| EMPA* | 4 | 0.38% | NA |  |
| EAWAG* | 4 | 0.38% | NA |  |
| **Gender** | **n** |  |  |  |
| Male | 798 | 75.43% | 3396 | 77.80% |
| Female | 254 | 24.01% | 967 | 22.20% |
| No answer | 6 |  | NA |  |
| **Age** | **n** |  |  |  |
| Birth year | *m*=1964, *sd*=8.2 |  | NA |  |
| No answer | 11 |  |  |  |
| **Discipline** | **n** |  |  |  |
| Social sciences and humanities | 294 | 27.79% | 1095 | 25.10% |
| Economics and business | 64 | 6.05% | 479 | 10.98% |
| Law | 50 | 4.73% | 312 | 7.15% |
| Natural sciences | 306 | 28.92% | 1058 | 24.25% |
| Medical and Health Sciences | 252 | 23.82% | 949 | 21.75% |
| Engineering and Technology | 64 | 6.05% | 417 | 9.56% |
| Other | 28 | 2.65% | 53 | 1.20% |
| **Nationality** | **n** |  |  |  |
| Switzerland | 497 | 46.98% | 2171 | 49.76% |
| Germany | 232 | 21.93% | In « others total» below |  |
| France | 61 | 5.77% | In « others total» below |  |
| Italy | 60 | 5.67% | In « others total» below |  |
| United States of America | 32 | 3.02% | In « others total» below |  |
| Others | 176 | 16.64% | In «others total» below |  |
| Others total | 561 | 55.02% | 2191 | 50.22% |
| No answer | 0 | 0% | 1 | 0.02% |

Table: Institution, age, gender, discipline and nationality of the sample (n=1058). The OECD discipline categories from our survey were reduced to the 7 categories used in the government statistics. * These institutions were not included into the statistical models as no institutional variables are available.
